# Seven Cases of Diphtheritic Croup—Two Aborted by Antitoxin and Five Cured by Antitoxin and Intubation

**Published:** 1899-06

**Authors:** R. M. Harbin

**Affiliations:** Rome, Ga.


### SEVEN CASES OF DIPHTHERITIC CROUP—TWO ABORTED BY ANTITOXIN AND FIVE CURED BY ANTITOXIN AND INTUBATION.*

BY R. M. HARBIN, M.D.,

ROME, GA.

CASE 1. Dewey F—, aged ten months, a well-nourised boy, was taken on September 15th, 1898, with symptoms of an ordinary cold and difficulty in swallowing. There was considerable hyperemia of the pharyngeal vault with some deposit of membrane on

* Read at meeting of Georgia State Medical Association, Macon, April, 1899.

each tonsil. Two days later hoarseness began and the next day dyspnea was well marked and the temperature ranged from 101 to 103 F. I was asked to see the child by Dr. W. D. Hoyt, on the 19th, at 9 A.M., and found an abundant deposit of grayish membrane on the tonsils and uvula and a great amount of swelling of the lymphatic glands of the neck. Breathing was very labored. Urine normal in quantity. This case furnished a perfect clinical picture of diphtheria. The usual remedies had been given and at 12 o'clock 1500 units of antitoxin (P. D. & Co.) was given and preparation was made for intubation. The throat was mopped with Loeffler's solution. The dyspnea remained about the same for six hours and in twenty-four hours the breathing became normal, the deposit on the tonsils having cleared away. No other treatment was given and the patient made a good recovery.

CASE 2. Annie T—, aged one year and one month, had sore throat on January 16th, 1898. I saw her on the 19th, and found an intense hyperemia of the pharynx and tonsils with a grayish deposit on each tonsil, with considerable swelling of the lymphatic glands of the neck. Temperature was 102 and pulse 130. A clinical diagnosis of diphtheria was made. There was some hoarseness on the 20th; there was slight dyspnea, indicating an invasion of the larynx. The usual remedies had been given and the room was vaporized from a kettle containing lime and turpentine. There being no abatement of symptoms 1500 units of antidiphtheritic serum (P. D. & Co.) was injected in the thigh and no other remedies were then given. In twenty-four hours the croup symptoms had disappeared and recovery was uneventful.

CASE 3. Adeline B—, aged three years eleven months and previous health good, had an attack of acute indigestion on November 2d, 1898, with temperature $101\frac{1}{5}$ and pulse 122. There was some hyperemia of the pharynx and tonsils, which later became more suspicious. During the two days following the temperature ranged from 99 to 102 and pulse from 106 to 122. On November 4th I was asked to see the case with Dr. Battey, and found the tonsils swollen and red with a slight deposit on the right tonsil resembling coagulated mucus and the glands of the neck were very much enlarged. The temperature was 101 and pulse 119, and urinalysis showed the absence of albumen. The patient was put upon a liquid diet and throat sprayed every four hours. For two days about the same set of symptoms continued and a clinical diagnosis of diphtheria was made. On the 6th hoarseness began and the next day at 4 P.M a severe paroxysm of dyspnea occurred. On the 8th at 1 A.M., 1500 units of antitoxin (P. D. & Co.) was given and six hours later nine ounces of urine was passed, having sp. gr. 1020 acid reaction and no albumen. At 12 A.M., there being no improvement of the symptoms 1500 more units of the lower grade of antitoxin was given. Obstructed breathing became greater and cyanosis developed, with pulse 120, intermittent and irregular, and temperature 100. At 5:30 P.M. intubation was done, producing instant relief with free expectoration. Some frozen milk was given and also an enema of raw egg and peptonoids. No urine was passed for twenty-four hours and then only five ounces. Urinalysis showed sp. gr. 1022, acid reaction and loaded with albumen and hyalin casts. The pulse varied from 120 to 130 and temperature remained under 100 and she was more or less stupid, slept well, had very little coughing and took nourishment fairly well, being supplemented by nutrient enemata. During the next twenty-four hours eight ounces of urine was passed having sp. gr. 1020, acid reaction and contained one-half per cent. of albumen. The quantity of urine now increased and albuminuria disappeared ten days after the operation. The tube was removed on the fifth day and improvement was slow. Eight days after administering the antitoxin an erythematous rash developed and lasted twenty-four hours. Convalescence was tedious and she became very emaciated, but ultimately made a good recovery.

CASE 4. H— C—, a healthy four and a half year old boy, was taken ill December 27th, 1898, two days after his arrival from Baltimore, with sore throat and vomiting, having a temperature of 102°. On the 29th there was some deposit on the tonsils, and on the 30th hoarseness began, and the next day dyspnea developed, and I was asked to see the case by Dr. J. C. Watts, of Cave Spring, Ga. On January 1st I found an abundant dirty, grayish membranous deposit on the tonsils with a tendency to bleed, and a very fetid breath. Breathing was very labored, temperature 99, pulse 120 to 130, very irregular, and urine scant, containing one-half per cent. of albumen. The diagnosis was evident, there being another case of diphtheria in the same family. At 2 P.M. 1500 units of antitoxin (P. D. & Co.) was given and intubation was done after some difficulty, with instant relief, and he took an hour's restful sleep. He took nourishment well and did well for fourteen hours, when without any premonition, heart paralysis came on after taking some frozen milk. Seeing him twenty minutes later I found him pulseless and unconscious. The tube was removed without any effort at resistance and strychnine and whisky were given hypodermically. Heart sounds were not discernible but reaction appeared in twenty minutes and the pulse at the wrist was 150 and very faint. Breathing was comparatively easy for a while, but in six hours it became necessary to reintroduce the tube. Strychnine gr. $\frac{1}{60}$, was given every four hours hypodermically, milk punches by the mouth and raw egg and peptonoids per rectum. The pulse remained irregular and intermittent for twenty-four hours, afterwards becoming more steady. One peculiarity was a profound toxemia with a normal temperature. Expectoration was free and easy. The urine increased in quantity and urinalysis showed less albumen than at the time of injecting the antitoxin. The tube was removed on the fourth day and his recovery was rapid, and no further disorder of the heart was noticeable, but the strychnine was kept up for two weeks.

CASE 5. Ruth B., a healthy, three-year-old girl, was taken with a croupy cough and symptoms of acute indigestion on November 27th, 1898. There was no redness of the fauces, and she developed no symptoms of importance until December 3d, at which time I was asked to see her with Dr. L. P. Hammond. There was complete aphonia, but had no symptoms of obstructed breathing and very slight changes in the temperature and pulse. There was no redness or deposit in the pharynx, but the case was viewed with suspicion. This condition continued until December 8th, when dyspnea developed somewhat paroxysmal in character. She was now very pale with pulse 150 and temperature 99, and urine scant, containing a trace of albumen. A clinical diagnosis of membranous croup with a probable diphtheritic infection was made and 2000 units of antidiphtheritic (P. D. & Co.) serum was given at 1 P.M. At 10 P.M. the labored breathing was greater, pulse 175 and temperature 99, with some cyanosis, and intubation was successfully performed, giving entire relief. December 9th, 10 A.M., takes nourishment well and expectorates freely, pulse 140, temperature 99, and urine sufficient in quantity, having sp. gr. 1030, acid reaction with no increase of albumen. The antitoxin showed no tendency to increase albuminuria. The tube was removed at the middle of the fourth day, and recovery was complete.

CASE 6. Annie T., aged five years and healthy, was taken with a croupy cough and aphonia on January 2d, 1898. Tonsils were red, swollen and contained a small deposit of membrane. A clinical diagnosis of diphtheria was made and the usual remedies applied. On January 5th, dyspnea developed, and on January 6th, after giving 1500 units of antitoxin (P. D. & Co.), intubation was performed by the assistance of Drs. Garlington and W. P. Harbin. Dypnea was entirely relieved, and she expectorated fairly well. There were unimportant changes in the temperature and pulse, and the kidneys acted well. She refused food altogether for two days, but by rectal feeding and coaxing she was made to take enough to sustain her. The tube was removed on the fifth day, and she made a good recovery.

CASE 7. Lucy W., aged three and a half years and healthy, had had tonsillitis three days before I saw her with Dr. T. R. Garlington on December 18th, 1897. There being the usual symptoms and albuminuria, a clinical diagnosis of diphtheria was made. Croupy symptoms developed the day before I saw her and dyspnea gradually increased, and on the 18th, 11 P.M., 1500 units of antitoxin (P. D. & Co.) was given, and intubation successfully performed. She was entirely relieved, expectorated freely and took nourishment well. The tube was removed on the fourth day, and her recovery was rapid.

As to the effects of antitoxin there can be no reasonable doubt but what it has an inhibiting influence on diphtheria, since there has probably been no other question in the medical profession so thoroughly studied by competent observers.

It has its best effect when given early, and shortens the course and lessens the severity of the disease. When used late in diphtheria its inhibiting effect is less marked, but still apparent and retards the absorption of toxins at the site of infection, which effect explains its favorable influence in intubation cases. Its good effect is noticeable where there is a streptococcus infection. When antitoxin is used in large quantities in cases exhausted by a previous illness causing anemia and albuminuria, its restraining effect on the kidneys is more liable to occur and its administration should be very guarded. As to the nature of the remedy little or nothing is known, but that does not hinder us from getting its good effect. The outer surface of the thigh is the place to be preferred for injecting the remedy and the parts should be scrubbed and rendered aseptic by a carbolic solution. The skin should be pinched up and the needle of the antitoxin syringe should be inserted horizontally, and by this manner the operation is rendered painless. The immunizing dose should be from 200 to 1000 units, and in severe laryngeal cases 1500 to 4000 units should be used. In weak, anemic and albuminuric cases I would never use over 2000 units.

For the successful performance of intubation a careful study of the details is very necessary. The tubes usually used, I think, are too short, as the longer are less apt to be coughed out, and become obstructed by detached membranes, and should preferably be made of hard rubber, as lightness of the tubes makes them more easily tolerated by the larynx.

I prefer the O'Dwyer set modified by Dr. Dillon Brown of New York, and have never had one coughed out. Before operating the tube should be secured with a braided silk thread. The child should be wrapped in a blanket and held by the nurse sitting in a straight chair. The gag is inserted and the mouth opened, but not too widely. The head must be held firmly in the median line and brought forward so as to make the axis of the larynx perpendicular to the horizontal plane of the tongue. The left index-finger is passed down to the epiglottis which is raised and the tube can be guided into the larynx by holding the introducer in the median line. Neglect of these details will defeat the success of the operation. When tube is in the larynx a moist rattling sound is heard and a quantity of mucus rises in the throat and the intervening wall between the tube and esophagus can be felt. The operation should occupy not more than ten seconds. The thread should be detached from the tube after half an hour, as it annoys the child no little.

The tube should be worn from three to seven days, and when expectoration gradually ceases, it can be safely removed. The dangers of intubation are few, being shock, apnea, and dislodging membranes rendering tracheotomy necessary. The successful issue depends very much on the after-treatment. The atmosphere of the room should be kept saturated with vapor from a kettle containing lime and turpentine, at a temperature of 65° to 70° F. One of the most difficult features of the after-treatment is feeding the patient, and I find most children refuse to take nourishment by the method recommended by Casselberry. Some refuse food altogether and should be nourished by the rectum. An unlimited amount of tact is required to get the cooperation of the child. It should be made to lie on its side with the head hanging slightly over the edge of the bed and a certain position is had in which food can be taken with comfort. Frozen milk and cracked ice can be best swallowed, and should be allowed if the child is sthenic; otherwise milk and soft cooked eggs should be given. Whenever the child has a paroxysm of cough, the head should be elevated. The throat should be sprayed only to correct fetor, as the child should be annoyed as little as possible, and no medicines should be given by the mouth.

The same procedure is required for removing as introducing the tube, and is more difficult.

### CONCLUSIONS.

1. Numerous statistics prove that antitoxin has lessened the mortality of diphtheria one-half.

2. The remedy is never toxic in its effects, and never causes or increases albuminuria, if properly used, and does not interfere with the use of other remedies.

3. The dose of antitoxin in laryngeal cases should be from 1500 to 4000 units, according to the age and condition of the patient.

4. In weak anemic and albuminuric cases its administration should be more guarded, as the restraining effect on the kidneys in Case 2 was due to a faulty administration.

5. Antitoxin favors resolution of the membranes in cases subjected to intubation, lessening the absorption of toxins, and renders an early removal of the tube safe.

6. Antitoxin has a favorable effect on the mixed infections, as in Case 3.

7. From a personal observation of three successful out of five tracheotomies and five consecutive successful intubations for croup, my preference is for intubation, except in rare cases.

