# Erratum

**Published:** 1899-07

**Authors:** 


ERRATUM.—Initials of Dr. Hutchins' name at head of his article in June issue should have been *M. B., not B. M.*

